# Clinical and Epidemiology Characteristics of COVID-19 Cases Detected During Mass Screening Campaign from July to October 2020 in Bujumbura, Burundi

**DOI:** 10.24248/eahrj.v6i2.689

**Published:** 2022-11-30

**Authors:** Edouard Nkunzimana, Jean Claude Bizimana, Adolphe Ndoreraho, Liesse Iteka, Pascal Butoyi, Ouedraogo Leopold, Joseph Nyandwi

**Affiliations:** a Ministry of Public Health and Fight against AIDS, National Public Health Institute, Bujumbura, Burundi; b Ministry of Public Health and Fight against AIDS, National Public Health Emergency Operation Centre, Bujumbura, Burundi; c World Health Organization, Country office, Bujumbura, Burundi

## Abstract

**Background:**

Coronavirus disease of 2019 (COVID-19) is an infectious disease caused by the Severe Acute Respiratory Syndrome Coronavirus 2 (SARS-COV-2 Virus). It was reported for the first time in Wuhan city, Hubei province of China. The first cases of COVID-19 in Burundi were identified on 31^st^ March 2020. Several signs and symptoms, including mainly; fever, dry cough, fatigue, myalgia, and dyspnea are the most prominent characteristics of the disease. The aim of this study was to provide description of the clinical and epidemiological characteristics of COVID-19 cases identified during the mass screening campaign conducted between July and October, 2020 in Burundi.

**Methods:**

We conducted a retrospective secondary analysis of data of clients to the mass screening campaign in Bujumbura city that was run between July and October 2020. Clients with complete data and tested for COVID-19 with Reverse Transcription Polymerase Chain Reaction (RT-PCR) were included in the study. Epi-Info 7.2.2.6 was used to perform descriptive and analytical statistics and Quantum Geographic Information System (QGIS) was used for cases mapping. Association between positive cases and independent variables such as sex, history of contact with confirmed COVID-19 case was measured using chi-square statistical test at a *p-value* of .05.

**Results:**

The study included 20,114 participants. 243 (1.2%) were tested positive for COVID-19. The mean age for confirmed cases was 33 (±15) years. The majority of cases (72.8%) were between 20 and 59 years of age and they were predominantly males (67.9%). 164 (67.5%) were symptomatic and cough was the most frequent symptom observed 109 (66.5%), followed by rhinorrhea 69 (42.1%). Fever was present in only 18 (11.0%) of symptomatic patients. Participants with a history of contact with a COVID-19 confirmed case (aOR=2.2; 95%CI [1.6-3.0]; *p-value* <.001), were more likely to be positive for COVID-19. Also, those who were coughing (aOR=1.47; 95%CI [1.06-2.05]; *p-value*=.023) and having sore throat (aOR=2.4; 95%CI [1.1-4.9]; *p-value=.02*) were more likely to test positive for COVID-19.

**Conclusion:**

This study revealed that a significant proportion (32.5%) of COVID-19 patients were silent carriers of the virus. Data highlighted that high proportion of cases were among the active age group and contacts with confirmed cases, and noted high proportion of asymptomatic cases at diagnosis. Measures including routine testing of asymptomatic contacts could contribute to tackling corona virus in Burundi.

## BACKGROUND

On 31^st^ December 2019, the World Health Organization (WHO), China Country Office was informed of cases of pneumonia with unknown aetiology detected in Wuhan City, Hubei Province of China. The Chinese authorities identified a new type of coronavirus, which was isolated on 7 January 2020.^1^ The WHO named this new coronavirus pneumonia COVID-19.^2^ The disease is highly infectious, and its main clinical symptoms include; fever, dry cough, fatigue, myalgia and dyspnea.^3^ Since the initial detection of COVID-19, the disease has spread globally and was declared a pandemic on March 11, 2020, by the WHO.^4^ Its main route of transmission in humans is through direct contact or air droplets, the transmission risk is higher within a span of one metre from the infected person.^5^ The case fatality rate is reported to be around 2.2%^6^, which is far lower than the rates for the previous two coronavirus epidemics that occurred in the 21^st^ century – namely; Severe Acute Respiratory Syndrome (SARS)-CoV in 2003 (10%) and Middle East Respiratory Syndrome (MERS)-CoV in 2012 (37%).^7^

In the initial stage of the pandemic, sub-Saharan Africa reported some of the lowest infection rates of COVID-19. Numbers began to rise in late March 2020, with confirmed cases increasing across the continent, however, this number may reflect a shortage of tests and testing facilities.^8^

The first cases of COVID-19 in Burundi were identified on 31^st^ March 2020 from 2 Burundians from Dubai and Kigali.^9^ Since then, the epidemic has resulted in 515 cases with 1 death (case fatality rate of 0.2%) as of October 6^th^, 2020 and more than three-thirds (75.9%) of the cases in Burundi were recorded in Bujumbura City.^10^ Different studies have shown scarcity of information on the description of clinical characteristics of COVID-19 patients. To the best of our knowledge, no study describing the clinical and epidemiological characteristics of COVID-19 patients was conducted in Burundi. This study was designed to provide description of the clinical and epidemiological characteristics of cases presenting to mass screening sites in Bujumbura City (ETS Kamenge, Source du Nil Hotel and Paroisse Kanyosha), and confirmed to be infected with SARS-CoV-2 by real time Reverse Transcriptase Polymerase Chain Reaction (RT-PCR).

## METHODS

### Study Design

We conducted a retrospective secondary analysis of data from patients presented at screening sites in Bujumbura city between July and October 2020. Socio-demographic and clinical data was collected using Open Data Kit (ODK) collect v1.29.2 and transferred to CARP platform.

### Laboratory Methods

### Sample Collection, Transport, and Storage

Oropharyngeal swab specimens were transported in viral transport medium from screening sites to the National Reference Laboratory. The specimen were refrigerated (2 to 8°C) before testing was performed on Abbott Instrument Systems.

### Sample Processing

Sample testing was performed with the Abbott Real Time SARS-CoV-2 assay developed by Abbott Molecular. Abbott Real Time SARS-CoV-2 assay is a real-time reverse Transcriptase (RT) Polymerase Chain Reaction (PCR) test intended for the qualitative detection of nucleic acid from SARS-CoV-2 in respiratory specimens collected by a healthcare worker, from individuals suspected of COVID-19.

Qualified and trained clinical laboratory personnel specifically instructed and trained in the techniques of real-time PCR and in vitro diagnostic procedures performed the Abbott Real-time SARS-CoV-2 assay.

### Study Variables and Measurements

In this study, we considered the RT-PCR result for COVID-19 as the dependent variable. The independent variables were socio-demographic information (age, sex), history of contact with COVID-19 confirmed patient, and clinical presentation (symptomatic or asymptomatic).

### Data Management and Analysis

We received data in Microsoft Excel 2016 from the emergency unit of Burundi Ministry of Health and AIDS control. Data was cleaned and transferred into Epi-Info 7.2.2.6 for re-coding and analysis of the variables to suit the study objectives. Descriptive statistics were used to summarise the data and results were presented as frequency and proportions in tables and charts. QGIS was used to perform the distribution of cases by district of residence. Descriptive statistics were generated for explanatory variables and chi-square test was used to determine the independent predictors of a positive test for COVID-19. Multivariable logistic models were developed to assess the association between explanatory variables and the outcome variable. Explanatory variables that were significant in the bivariate analysis at *p<.20* were considered candidates for the multivariable logistic models. The final multivariable logistic models were developed using backwards elimination of the explanatory variables with *p values <.05.*

### Ethical Consideration

Permission to carry out the study was sought and obtained from Burundi Ministry of Health and Fight against AIDS. Ethical clearance was obtained from Burundi National Ethical committee (reference number: CNE/32/2021). To ensure confidentiality, participants' identifying information was not included in the dataset used in statistical analyses. This analysis was based on secondary data collected during screening campaigns, therefore, participants consent was not sought.

## RESULTS

### Socio-demographic Characteristics of Screened Patients and Confirmed Cases

A total of 20,114 participants were included in the analysis. Their mean age was 34 (±14) years. The Majority were males (14,605, 72.6%). A total of 7,636(38.0%) participants were screened at Source du Nil Hotel which is located in the Centre District of Bujumbura City. About 17,882(88.9%) participants were residents of Bujumbura City while 2,232(11.1%) were residents of other provinces especially around Bujumbura City (Bujumbura-4.6%, Bubanza-4.5%). Health professionals constituted 420(2.1%) of the participants. Among those screened, 10,364(51.5%) participants were symptomatic while 3,134(15.6%) had had contact with COVID-19 confirmed cases.

Two hundred and forty-three (243) cases were confirmed by laboratory RT-PCR test, which gives a positivity rate of 1.2%. The mean age for confirmed cases was 33 (±15) years. The prevalence of COVID-19 among male participants was 1.1% and 1.4% among females. Among health care workers, the prevalence of COVID-19 was 1.7%. Majority cases were from residents of Northern Health District with 101(41.6%), followed by Southern District 70(28.8%) and Central District 55(22.6%) of Bujumbura City (Figure 2).

### Clinical Characteristics of Confirmed COVID-19 Cases

**FIGURE 1:**
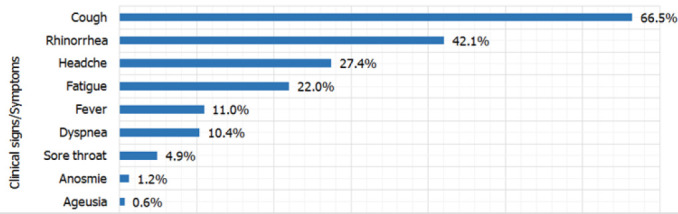
Clinical Characteristics of Confirmed Cases

About 164(67.5%) were symptomatic. Cough was the most common symptom 109(66.5%), followed by rhinorrhea, 69(42.1%), headache 45(27.4%), fatigue 36(22.0%), fever 18(11.0%), shortness of breath-dyspnea 16(10.4%), sore throat 8(4.9%), loss of smell-anosmia 2(1.2%) and lack of the sense of taste-agueusia 1(0.6%) (Figure 1). Eleven patients (4.5%) had chronic diseases such as hypertension, diabetes and Human Immunodeficiency Virus (HIV). Contact with COVID-19 confirmed case was declared in 24.3% of confirmed cases (Table 1).

**FIGURE 2:**
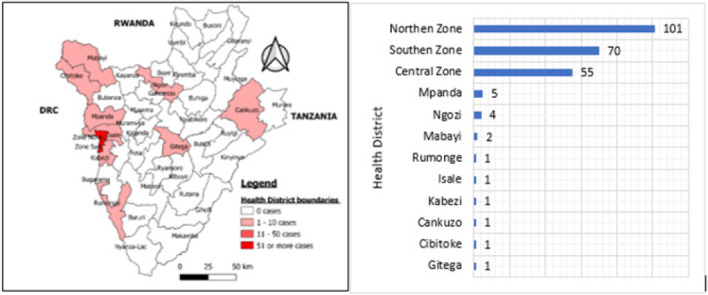
Distribution of Confirmed Cases by Health District of Residence, Bujumbura, July to October 2020

**TABLE 1:** Characteristics of screened persons from July to October 2020, Bujumbura, Burundi

Characteristics	All screened persons		Confirmed cases	
	Number	%	Number	%
**Site of screening**				
ETS Kamenge	7608	37.8	100	41.2
Hñtel Source du Nil	7636	38.0	78	32.1
Paroisse Kanyosha	4870	24.2	65	26.7
**Sex**				
Male	14605	72.6	165	67.9
Female	5509	27.4	78	32.1
**Age**				
Mean (SD)	34	(14)	33	(15)
<5	269	1.3	7	2.9
5-9	407	2.0	4	1.6
10-19	2237	11.1	35	14.4
20-29	5295	26.3	63	25.9
30-39	5121	25.5	54	22.2
40-49	3793	18.9	46	18.9
50-59	2921	14.5	14	5.8
≥60	71	0.4	20	8.2
**Province of residence**				
Bujumbura Mairie	17882	88.9	226	93.3
Bujumbura	931	4.6	2	0.8
Bubanza	896	4.5	5	2.1
Other provinces	405	2.0	10	4.1
**Health professional**				
Yes	420	2.1	7	2.9
No	19694	97.9	236	97.1
**Contact history**				
Yes	3134	15.6	59	24.3
No or unknown	16980	84.4	184	75.7
**Symptomatic**				
Yes	10364	51.5	164	67.5
No	9750	48.5	79	32.5

### Factors affecting the COVID-19 RT-PCR Results among Screened Persons

This section presents factors found to be independently associated with RT-PCR positive results after adjustment of factors that were significant at *p-value <.2* in bivariate analysis (Table 2). Persons who had contact with a COVID-19 confirmed case (aOR=2.20; 95%CI [1.62-2.98]; *p-value <.001*) were two times more likely to be positive for COVID-19 compared to those with no history of contact. Also, coughing (aOR=1.47; 95%CI [1.06-2.05]; *p-value=.023*) and having sore throat (aOR=2.35; 95%CI [1.14-4.85]; *p-value=.021*) were statistically associated with COVID-19 RT-PCR positive result (Table 3).

**TABLE 2:** Association between some Selected Variables and the Covid-19 RT-PCR Positive Results, Bujumbura, July to October 2020

Variables	Covid-19 status						
	Positive		Negative		OR	[95% CI]	P-Value
	n	(%)	n	(%)			
**Age group**							
<10	11	(1.6)	665	(98.4)	Ref.		
10-19	35	(1.6)	2202	(98.4)	1.0	[0.5-2.0]	.999
20-29	63	(1.2)	5232	(98.8)	0.7	[0.4-1.5]	.433
30-39	54	(1.1)	5067	(98.9)	0.6	[0.3-1.3]	.256
40-49	46	(1.2)	3747	(98.8)	0.7	[0.4-1.5]	.485
≥50	34	(1.1)	2958	(98.9)	0.6	[0.4-1.4]	.393
**Sex**							
Female	78	(1.4)	5431	(98.6)	1.3	[0.9-1.6]	.113
Male	165	(1.1)	14440	(98.9)			
**Contact history**							
Yes	59	(1.9)	3075	(98.1)	1.8	[1.3-2.3]	<.001ⱡ
No/unknown	184	(1.1)	16796	(98.9)			
**Symptomatic**							
Yes	164	(1.6)	10200	(98.4)	2.0	[1.5-2.6]	<.001ⱡ
No	79	(0.8)	9671	(99.2)			
**Fever**							
Yes	18	(2.3)	759	(97.7)	2.0	[1.2-3.2]	.007ⱡ
No	225	(1.2)	19112	(98.8)			
**Cough**							
Yes	109	(1.7)	6161	(98.3)	1.8	[1.4-2.3]	<.001 ⱡ
No	134	(1.0)	13710	(99.0)			
**General weakness/fatigue**									
Yes	36	(2.0)	1746	(98.0)	1.8	[1.3-2.6]	.002 ⱡ
No	207	(1.1)	18125	(98.9)			
**Headache**							
Yes	45	(1.8)	2508	(98.2)	1.6	[1.1-2.2]	.008 ⱡ
No	198	(1.1)	17363	(98.9)			
**Sore throat**							
Yes	8	(3.2)	244	(96.8)	2.74	[1.25-5.35]	.010 ⱡ
No	235	(1.2)	19627	(98.8)			
**Dyspnea**							
Yes	17	(1.7)	960	(98.3)	1.5	[0.9-2.4]	.159
No	226	(1.2)	18911	(98.8)			
**Runny nose**							
Yes	69	(1.4)	4834	(98.6)	1.2	[0.9-1.6]	.164
No	174	(1.1)	15037	(98.9)			
**Anosmia**							
Yes	2	(3.3)	58	(96.7)	2.8	[0.7-11.7]	.359
No	241	(1.2)	19813	(98.8)			
**Comorbidity**							
Yes	11	(1.4)	787	(98.6)	1.2	[0.6-2.1]	.756
No	230	(1.2)	19084	(98.8)			

## DISCUSSION

The results from this study highlighted the COVID-19 RT-PCR positivity rate of 1.2% from July to October 2020.

This rate is lower than what is recorded in WHO African Region (9.0%).^11^ The prevalence (1.7%) of COVID-19 among health care workers in this study is lower than what was observed among health care workers in a University Teaching Hospital in Central Italy (2.7%).^5^ This difference may be explained by the fact that this study's participants voluntarily reported to the screening site. The mean age of COVID-19 confirmed cases was 33 (±15)years which is higher than that of the general population (21.3 years).^14^ Comparing to other studies, this mean age was lower than the mean age of patients in Lagos, Nigeria (46.2 years)^15^ and in Liaocheng, China (42 years).^7^ Children under 5 years of age and those aged 5 to 10 years, respectively, accounted for 2.9% and 1.6% of confirmed COVID-19 cases in this study. This shows that children are at low risk of getting infected by COVID-19 considering that the proportion of less than 5 years of age in general population is 17.9%.^14^ However, it remains unclear why children are less affected by COVID-19 than older individuals, evidence suggests that this could be due to differences in their immune system function.^17^ Majority (72.8%) of cases were among participants aged between 20 to 59 years which is similar to findings from studies conducted in Nigeria and Jordan.^15,18^ The higher proportion of COVID-19 cases recorded among economically active age groups suggests potential impact of socio-economic or work-related activities.

Clinically, 67.5% of confirmed cases were symptomatic. Our finding is different from the findings of a study conducted in Kuwait were only 41.0% of the participants were symptomatic.^19^Another study conducted in Nigeria reported that 33.0% of COVID-19 confirmed cases between the month February and June were symptomatic^20^, this is lower than this study's findings. However, a descriptive study conducted in Lagos, Nigeria found out that nearly all their study's participants (89.6%) were symptomatic^15^, this observed difference could be due to the fact our study was a health centre based study.

Clinical and Epidemiology Characteristics of COVID-19 Cases www.eahealth.org

A study conducted on COVID-19 patients admitted in the quarantine centre at King Abdullah University Hospital in Jordan between March 16 and May 21, 2020 showed that 58.0% of the participants were symptomatic.^18^ According to WHO, the main clinical presentation of COVID-19 cases are; fever, cough, general weakness/fatigue, headache, myalgia, sore throat, coryza, dyspnoea, anorexia/nausea/vomiting, diarrhoea and altered mental status.^21^ In our study, cough was the most frequent symptom, accounting for 109 (66.5%) cases, followed by rhinorrhea, (42.1%), headache (27.4%), fatigue (22.0%), fever (11.0%), dyspnea (10.4%), sore throat (4.9%), anosmia (1.2%) and ageusia (0.6%). The common clinical features observed in our study are similar to features observed by other related studies.^15,20^ Although fever was found to be the 5^th^ most observed symptom in our study, numerous other studies reported fever as the most prevalent symptom.^7,17,19,20,22^

This may be explained by the fact that public messages inviting people for early screening were sent and those who responded to the invite were not necessarily sick. Similarly to our findings, cough was found to be the most common symptom in a study conducted in Jordan between March 16 and May 21, 2020.^18^ Our study also showed that patients presented at screening sites with cough were more likely to test positive for COVID-19 (aOR=1.47; 95%CI [1.06-2.05]; *p-value=.023*). Although relatively small in proportion, patients with sore throat were also more likely to test positive for COVID-19 and this is consistent with available evidence.^15,18–20^

Eleven patients (4.5%) had chronic diseases such as hypertension, diabetes and HIV. This was low compared with other studies conducted elsewhere.^7,15,19^ For example, hypertension (17.6%) and type II diabetes mellitus (10.2%) were the most common comorbidities experienced at King Abdullah University Hospital in Jordan.^18^ Our study noted that patients with a history of contact with a COVID-19 confirmed case were more likely to be positive for COVID-19 compared to those with no history of contact. This is consistent with scientific evidence from studies conducted elsewhere.^5,15,23^ This explains that there was an active human to human transmission during that period. Then, although only 24.3% of the study participants were asymptomatic, it is known that they can be infective.^23^ Hence, it is important to identify asymptomatic cases as quickly as possible through testing of the contacts of each confirmed case, so that the spread of the virus can be controlled.

## CONCLUSION

In conclusion, this study revealed that a large proportion of COVID-19 patients (32.5%) were silent carriers of the virus. Economically active age groups were more likely to be infected with COVID-19, and the most occurring symptoms were; cough, rhinorrhea, and headache, which are similar to seasonal flu symptoms. The study also highlighted the high proportion of asymptomatic cases at diagnosis and among contacts with confirmed cases. Evidence from this study will be useful by policymakers and stakeholders in the health and other sectors in contextualising public health planning, policy and response as well as facilitating scientific activities in the country. Following the study's findings, measures such as case finding protocols should include routine testing of asymptomatic contacts.

### Study Limitations

The study has several limitations which are mainly related to the methodology used. Part of this research, should be considered. First, the external validity of the study to the community of Bujumbura cannot be retained since the data used was obtained from volunteers who visited the mass screening sites and these probably were suspecting themselves to be infected with COVID-19 and thus their willingness to test for the disease. Secondly, we were unable to determine the exact period of infection. since patients were discovered during the mass screening campaign, it is possible that they acquired the infection several days before the detection test.
